# Determining the Factors that Impede or Facilitate the Utilization of Telemedicine (Video Visits) for Geriatric Patients

**DOI:** 10.1089/tmr.2022.0018

**Published:** 2022-08-24

**Authors:** Onyeche Oche, Richard Dobyns, Cory Lin, Korey A. Kennelty

**Affiliations:** ^1^ College of Pharmacy, University of Iowa, Iowa City, Iowa, USA.; ^2^ Department of Family Medicine, University of Iowa, Iowa City, Iowa, USA.; ^3^ Carver College of Medicine, University of Iowa, Iowa City, Iowa, USA.

**Keywords:** barriers, COVID-19, facilitators, geriatric, older adults, telemedicine

## Abstract

**Objective:**

The objective of the study was to evaluate the barriers and facilitators of telemedicine utilization experienced by geriatric patients at the University of Iowa Family Medicine Clinic and selected Senior Living Communities in Iowa City, to inform recommendations for improving the telemedicine delivery process for older adults.

**Methods:**

The study population was elderly patients (65–85 years old) living independently, and in long-term care facilities, who received health care using telemedicine during the period of the study from March to July 2020. A Mixed Methods study design was utilized with qualitative data collected through semistructured telephone and Zoom interviews and quantitative data through surveys.

**Results:**

A total of 33 study participants (*n* = 33) were interviewed or surveyed, including 3 patients (*n* = 3), 4 caregivers (*n* = 4), 19 physicians (*n* = 19), 5 medical assistants (*n* = 5), and 2 schedulers (*n* = 2). The results showed that geriatric patients and their caregivers, as well as health and nonhealth care personnel experience barriers, including difficulty navigating technology, privacy concerns, and lack of technical support; and facilitators, such as customer service support and having protocols to guide patients on telemedicine use.

**Conclusion:**

Geriatric patients face certain barriers and facilitators (self-identified or identified by their caregivers, physicians, and other health and nonhealth care personnel) that can make it either more or less difficult for them to maximize the benefits of telemedicine. As a result, health systems should consider older adults' needs and preferences when implementing telemedicine systems in outpatient settings.

## Introduction

In the United States, the use of telemedicine (defined by the Centers for Disease Control and Prevention as “using electronic information and telecommunication technology to get health care while practicing social distancing”) increased significantly in both quantity and frequency in the early days of the COVID-19 pandemic (March to July 2020) compared with previous years.^1^

Among the elderly, a 2020 survey by the University of Michigan National Poll on Healthy Aging (NPHA) showed a 26% increase in the percentage of older adults (50–80 years) who participated in telemedicine visits between May 2019 to June 2020.^2^ The increase was due largely to the safety concerns associated with contracting COVID-19 at in-person hospital visits, but older adults also benefitted from the efficiency and convenience of using telemedicine.^3^ In spite of this increase in telemedicine use, however, studies have shown that older adults may not have the ability to conduct video visits because of age-related disabilities or technology inexperience.^4^ Other barriers to telemedicine use for older adults have been associated with design and interface issues, lack of devices, concerns about privacy and cost, lack of technology support, and inability to access the internet.^5–8^

On the other hand, studies have shown that home telehealth services can be beneficial to older adults who have chronic conditions and age-related impairments because they help to lower costs of care, increase the quality of life, and promote independent living.^8^ One study, in particular, found telemedicine to be particularly advantageous in chronic disease management by reducing hospital admissions or readmissions, length of hospital stay, emergency department visits, and mortality.^9^ In another study that linked telemedicine acceptance in older adults to facilitating factors, previous positive telemedicine experience, technical knowledge and support, increased access to care, decreased cost, recommendations by physicians, and convenience were reported as telemedicine facilitators.^10^ Altogether, barriers and facilitators can determine the extent to which older adults find telemedicine useful and how much they are willing to use it.

As the population of older adults increases (by 2030, 1 in 5 Americans is projected to be 65 and over),^11^ and the threat of future pandemics remains on the horizon, it is important to consider the telehealth care needs of older adults^6^ who are at risk for conditions requiring chronic care support.^12^ One study reports that older adults will be the vanguard of geriatric telehealth if only we would listen to what they had to say without any preconceived notions.^13^ Yet, while much has been studied about technology use in older adults, not much is known about the self-reported telemedicine experiences of the older adults themselves.^14^ In addition, the barriers and facilitators faced by their caregivers, and health care workers as they made adjustments to care giving during the pandemic has not been studied extensively.

This study fills the gap by not only focusing on the barriers and facilitators of telemedicine from the point of view of the older adult patients, but also assessing the experiences of others involved in the patients' care to get several perspectives on this critical issue. In addition, it goes a step further to propose recommendations for improving the telemedicine system based on the experiences of patients and those involved in patient care. This approach is useful in ensuring that members of this vulnerable population are not left behind in the face of dramatic technological changes in the current health care delivery landscape.

At the onset of the pandemic, telemedicine video visits were frequently scheduled at the University of Iowa Hospitals and Clinics for our geriatric patients, even though some were ultimately converted to telephone visits. This switch from video to telephone raised concerns about the process of providing high-quality care with telemedicine video, prompting the need to identify the facilitators and barriers that were being experienced by our patients, to inform recommendations for improving the telemedicine delivery process.

Guided by a psychological theory (the Theory of Planned Behavior),^15,16^ which shows how attitudes, subjective norms, and perceived behavioral control can lead to an individual's behavioral intentions, we developed questions and conducted interviews with patients, their caregivers, physicians, patient schedulers, and medical assistants (MAs) over a 4-month period, to determine factors that impeded or facilitated telemedicine video use. Cross-sectional surveys were also sent to physicians at the Geriatric Assessment clinic to understand their experiences with telemedicine and what they perceived were barriers and facilitators among their patients. Some interesting findings from the surveys were subsequently used to develop interview questions to better understand the telemedicine experiences of physicians.

## Materials and Methods

### Institutional review board exemption

The University of Iowa's Institutional Review Board (IRB) approved the study as a quality improvement project, obviating the need to obtain patient consent.

### Study design and setting

We conducted our study at the University of Iowa Hospitals and Clinics in collaboration with Senior Living Communities (SLCs) in the Iowa city area. The specific location for the study was the Geriatric Health Assessment Clinic (within the Family Medicine Clinic), which utilizes team-based care in the evaluation and treatment of geriatric patients. A mixed methods study design was employed, with quantitative data collected through provider surveys, and qualitative data through semistructured telephone and Zoom interviews. This was followed by another semistructured interview to further explain the provider survey findings.

### Recruitment and participants' characteristics

Fifty-seven physicians at the Family Medicine and geriatric clinic of the main campus of the University of Iowa were contacted about the study by a researcher, R.D. After identifying the names of the physicians who had 4 or more telemedicine video visits in the study period by running reports, surveys were sent out to the 23 physicians who met the criteria. Although there was an increase in the total number of telemedicine visits (geriatric and younger patients) seen by the selected providers in the March to July 2020 time period, we were only interested in the telemedicine video visits of our geriatric patients. We decided on four patients because we had very few telemedicine visits for our geriatric patients before COVID (in 2019) and our health system was only seeing acute visits for the first 2 months of the pandemic (March to April 2020). Furthermore, many of our providers were pulled into in-patient care during this period and were not having routine visits as usual.

Out of the 23 physicians, 18 responded fully to all survey questions, giving a survey response rate of 78.3%.

The recruited providers were also contacted by personalized emails sent out by the project director, requesting permission to interview their patients and MAs. After obtaining permission to interview, letters were sent out to only geriatric patients whose physicians had carried out more than four telemedicine video visits during the study period. The patients' letters contained information requesting their participation and a $15 payment for completing the interviews. Schedulers were recruited through emails sent to the head of the scheduling unit.

### Sampling

We ran reports to determine the number of telemedicine visits each patient had. Following this review, we stratified our provider population (57 geriatric physicians) based on telemedicine video use, into high and low utilization groups. High utilization providers were identified as those with ≥4 telemedicine video visits from March 2020 to July 2020 while low utilization providers had <4 telemedicine video visits in the same study period. From this population, we purposively sampled 23 physicians who had 4 or more telemedicine video visits to be interviewed.

### Pretesting

We pretested our data collection instruments (the Qualtrics surveys and semistructured interviews) to refine them, test comprehension of the questions, and reduce measurement errors. The Qualtrics survey was pretested with one of our physician researchers while the semistructured interview was pretested with a scheduler at the University of Iowa Hospitals and Clinics. After pretesting, questions that were identified as difficult to understand were rephrased and items that could potentially confuse interviewees were taken out. To increase reliability and precision of our measurement, we ensured that we had several items to measure a construct as recommended by Kimberlin and Winterstein.^17^

### Data collection

#### Quantitative data

Provider surveys consisting of 20 questions measuring the Theory of Planned Behavior constructs, such as attitudes, subjective norms, and behavioral intentions were administered using Qualtrics. Questions 1 to 15 utilized a 7-point Likert scale, Question 16 required a “yes/no” response, whereas questions 17 to 20 were open-ended to allow the participants the freedom to fully express their opinions. We used different formats to encourage honest and truthful responses as opposed to convenient responses.

#### Qualitative data

Qualitative data were collected using semistructured interviews administered by O.O. and K.A.K. over the phone or Zoom software to 15 of the 33 study participants, including 3 patients (*n* = 3), 4 caregivers (*n* = 4), 1 physician (*n* = 1), 5 MAs (*n* = 5), and 2 schedulers (*n* = 2). Verbal consent was obtained from patients before each interview was recorded and interviews lasted between 30 and 45 min depending on the number of follow-up questions asked.

### Data analysis

Thematic analysis was done by O.O. using a deductive approach, which entailed a close examination of the data to identify common themes. Specific steps were also followed based on the recommendations of Braun and Clark,^18^ including familiarization with the data, coding, generating themes, reviewing themes, defining and naming themes, and writing the report.

## Results

Our findings on telemedicine barriers and facilitators are reflected in the constructs of the theory of planned behavior, which relate the intention to carry out a behavior (in this case, the use of telemedicine) to the disposition of the user toward it (attitude), what they feel their significant others think about it (subjective norm), and how they feel they are supported to use it (perceived behavioral control). For instance, one of the technologically savvy older adult patients had a good attitude toward using telemedicine because it was easy for him to use. This was unlike an older caregiver who felt that more training was needed to feel competent about using telemedicine (lack of perceived behavioral control). In addition, knowing that a significant other was also using telemedicine (subjective norm) was a telemedicine facilitator for a couple. Our findings are shown below:

### Qualitative findings from semistructured interviews

Representative quotes for the barriers and facilitators are used in this study to include the voices of the participants and provide a basis for how we reached our conclusion.

Patient-reported barriers were primarily technological, and largely related to navigation difficulties when attempting to download the telehealth application. Other barriers included inaccurate messaging received about meeting the requirements for a telemedicine visit and a mismatch between health conditions and telemedicine video use.

Barrier 1: Patient's low digital literacy level

Well, a lot of the older people just don't understand it…, they'd have to have somebody around them that knew what they were doing.

Barrier 2: Inaccurate messaging about telemedicine visit requirements

I think they sent a link, let me take that back, the fellow, and I don't know if it was a pharmacy guy, that said [him], because he's in Illinois, he can't do telemedicine.

Barrier 3: Condition deemed inappropriate for telemedicine video visits

The particular kind of issue that I wanted to talk about was one that wasn't very amenable to doing it by telemedicine. He really would've needed to be able to manipulate my leg to figure out what was going on.

Facilitators included patient's technological skill level, patient's experience with using other software for video communication, and caregiver technical assistance.

Facilitator 1: Patient's high technological skill level

And I think that because we're technologically savvy, it really wasn't a big step for us. I mean, she and I, we each have and operate separate computers and that's most of the time the way we talk to each other.

Facilitator 2: Patient's experience with using other software for video communication

We've had Zoom meetings with a family where we've all sat down at dinner together at different places. And we've done that with friends.

And I know lots of people who did that [used other video technology software]. And I think that generally makes them familiar with communicating by an audio-visual method.

Facilitator 3: Technologically savvy caregiver

No, I got him [the physician] right on. See, we do that here at work. We have laptops now that we're doing this COVID thing, we have laptops and we get on all the time, with our Microsoft teams and stuff.

### Formal caregiver-reported barriers and facilitators

Barrier 1: Lack of information about provider wait time

The main thing is I would have a set time to get on with him, and if he was running behind a lot of times, I didn't know. I would be waiting at the computer for him to come on.

Facilitator 1: Straightforward log in process after first attempt

After the first initial time it wasn't too bad then after that it would just automatically open up when I clicked on the link.

### Informal caregiver-reported barriers and facilitators

Barrier 1: Help desk lines are not always helpful

Oh, man, those help desk lines. I never think those work well. I guess it just depends on who's manning or womaning the desk.

Facilitator 1: Provider nonmasking showed concern for deaf patients

And by the way, the positive for my father is that he's basically deaf and he does have two hearing aids, but he reads lips. And so, when you've got a mask on in the office, it's very hard for him to understand what's going on. But when he could see Dr. D's face right in the screen, that was easier for him.

MA-reported barriers included difficulty reaching patients residing in a facility, uneven customer service support from the IT help desk, a lack of awareness by patients that the MAs would call before their appointment, and lack of telemedicine training. A facilitator included being able to link patients with the customer service support.

Barrier 1: Delays in treatment due to patient's unawareness of MAs's phone call

If the patients would know that the MA or the nurse is going to call them 10, 15 minutes before the appointment. Because every time I try to call, they don't answer sometimes.

Barrier 2: Lack of telemedicine training

The only time it is difficult, and we have a problem I think is when the patients can't figure it out and we're on the phone with them asking if they need help and we're not quite prepared on how to tell them what to do.

Facilitator 1: Customer service support for patients

…but we do also have a customer support number that we can give them in case they run into an issue.

Physician-reported barriers included device malfunctions, which could require switching to secondary devices during a visit and the patient's functional ability, while a facilitator included removal of perceived threats (PVs) for certain patients.

Barrier 1: Barriers related to patients' functional ability

I think it's hard because these are end stage Parkinson's and they're having trouble communicating, getting the words out. And so, they feel the pressure.

Facilitator 1: Removal of PV for older adults with Parkinson's disease:

And I had some Parkinson's patients who, for whatever reason, I don't think they like me in their space as much, and so talking to a computer was less threatening and they seemed to open up a little bit. So, we have the full range of experience.

Scheduler-reported barriers included telehealth application download difficulty and nonuser-friendly patient health management tool (MyChart) updates, while facilitators included protocols to guide patients on the basics of telemedicine use and patients' use of devices (laptops or desktops) not requiring telehealthConnect app download.

Barrier 1: App download difficulty

That's where we're losing a lot of patients. I mean, honestly, geriatric or not, that part is confusing for people to figure out how to download that third party app outside of MyChart.

Barrier 2: Nonuser-friendly patient electronic record (MyChart) updates

When MyChart updated kind of in the middle of COVID, and once it updated, it was very unuser-friendly, it was ridiculous for everybody. They used to be able to just to figure it out, it was a super easy thing to do. And then once it updated, it just was hard for everybody.

Facilitator 1: Designing a stepwise protocol for patients

Honestly, it's like an A to Z. You know what they're going to look at when it first comes on and then once they click on that, you just need to go down. It's like teaching my toddlers. You have to go step by step by step, because if they skip a step, it's not going to work at the end.

Facilitator 2: Using laptop or desktops that do not require the telehealthConnect app.

And honestly, I think a lot of geriatric patients, they're going to do it from their desktop or from a laptop anyway. I don't think a lot of them we had to talk about the telehealthConnect app.

### Quantitative findings from providers' surveys

The results of the closed-ended section of the providers' surveys (questions 1 to 15) are shown in Table 1 below. The numbers indicate the number of providers who selected a particular option. Overall, there were more favorable responses for telemedicine video than phone, indicating that providers were more inclined to use telemedicine video over phone. Interestingly, there appears to be an even split between the number of providers who “strongly agreed” and those who “somewhat agreed” that the hospital's telemedicine technology was easy to use. This is an area that could be further explored to determine what can be done to address specific areas of difficulty for those who “somewhat agreed.” However, more providers “strongly agreed” than those who “somewhat agreed” that telemedicine is beneficial to geriatric patients.

**Table 1.** Summary of Providers' Responses

		Strongly disagree	Disagree	Some what disagree	Neutral	Somewhat agree	Agree	Strongly agree
1. The hospital encourages us to use telemedicine for our geriatric patients.	Vid	2	2	1	1	4	1	1
	Phone		3	3	1		1	
2. The hospital's telemedicine technology is easy to use.	Vid				1	5	4	
	Phone			1		1	3	5
3. Currently, if there are problems when using telemedicine with my geriatric patients, I can contact people who will respond readily.	Vid	2	1	2	1	4		
	Phone	2	1	2	1	2	1	1
4. Telemedicine use for geriatric patients is widely accepted across the institution.	Vid		1	1	3	2	2	1
	Phone		2	2	4	1	1	
5. Reimbursement for geriatric telemedicine visits should be equal to in-person visits.	Vid			1		1	3	5
	Phone			1	2	3	2	2
6. The quality of care provided for my geriatric patients through telemedicine matches that provided through in-person visits.	Vid			1	3	2	3	1
	Phone			2	5		2	1
7. My geriatric patients can benefit from telemedicine.	Vid					1	4	5
	Phone					2	4	4
8. My geriatric patients and I understand each other well when telemedicine is used.	Vid				1	2	4	2
	Phone				1	4	3	1
9. I feel comfortable giving diagnoses to my geriatric patients over telemedicine.	Vid				2	1	3	4
	Phone				2	4	2	2
10. I have reservations about my geriatric patients' privacy during telemedicine use.	Vid	2	5	1	1	1		
	Phone	2	4	1	2	1		
11. I can adequately assess my geriatric patients' illnesses with telemedicine.	Vid			2		3	3	2
	Phone			2		4	3	1
12. My geriatric patients or their caregivers need additional help operating the technology used in telemedicine.	Vid		1			2	6	1
	Phone		3	3	1	2	1	
13. I will recommend telemedicine use to my geriatric patients.	Vid				3	1	4	2
	Phone		1	2	1	1	4	1
14. Telemedicine use for geriatric patients is sustainable in the long run, even after COVID.	Vid		1		2		4	3
	Phone		2		3	1	2	2
15. I will use telemedicine for my own geriatric patients in the future.	Vid		1		2		4	3
	Phone		2		2	1	3	2

One response is a test response.

MA, medical assistant.

Expectedly, many providers, when asked if they could adequately assess their patients' illnesses with telemedicine did not “strongly agree,” making a complete conversion from in-person to telemedicine visits quite unlikely in the future. However, they were in agreement about recommending telemedicine to their patients.

The findings from the providers' surveys were used to design a semistructured questionnaire to obtain qualitative data to provide a deeper understanding of providers' perspectives on the barriers and facilitators that older adults experience with telemedicine video visits.

## Discussion

Our study identified several barriers and facilitators of telemedicine utilization experienced by geriatric patients, their caregivers, and other personnel at the University of Iowa Family Medicine Clinic as well as selected SLCs in Iowa City. These barriers included, but were not limited to, difficulty with using technology, IT support issues, lack of proactiveness about accommodating a patient's functional status, and a lack of training on how to use telemedicine efficiently. Facilitators, on the other hand, included being cognizant of a patient's functional status during the telemedicine visit, providing protocols to guide patients' use of telemedicine, technical assistance through caregivers, and access to devices (e.g., laptops, desktops) that did not require downloading the telehealth app.

Our findings are reflected in the constructs of the theory of planned behavior, which relate the intention to carry out a behavior (in this case, the use of telemedicine) to the disposition of the user toward it (attitude), what they feel their significant others think about it (subjective norm), and how they feel they are supported to use it (perceived behavioral control).

For instance, one of the technologically savvy older adult patients had a good attitude toward using telemedicine because it was easy for him to use. This was unlike an older caregiver who felt that more training was needed to feel competent about using telemedicine (lack of perceived behavioral control). In addition, knowing that a significant other was also using telemedicine (subjective norm) was a telemedicine facilitator for a couple.

While some studies have investigated the barriers and facilitators of telemedicine in the older adult population, what made this study unique is that we sought multiple perspectives and conducted it in the early days of the pandemic, where there were a lot of upheavals and adjustments due to how telemedicine was rapidly introduced, causing providers and patients to adopt and use it quickly,^14^ and giving older adult patients very little choice in terms of their preferred telemedicine experience. In addition, our study built on previous studies by providing recommendations about how the telemedicine care delivery process could be improved for older adult patients from the perspectives of the patients and other stakeholders.

In a recent study conducted by Mao et al,^14^ the researchers sought to understand the barriers to telemedicine video visits in older adults to improve the experience of virtual visits as well as access to remote care. They found the largest barriers to be difficulty with technology, difficulty hearing, linguistic barriers, and an unwillingness to meet with providers virtually.

Although linguistic barriers and an unwillingness to meet with providers virtually were not findings from our study, difficulty with operating technology, which was a finding in our study, is consistent with prior literature on the telemedicine barriers faced by older adults.^4^ However, older adults are not a monolith and do not all have similar experiences with technology^6^ as reflected by some of our findings from the study. Effort should be made, therefore, to ensure that interventions are tailored to the needs of the patients.

In addition, other findings such as a patient's previous experience with using other software for video communication are consistent with literature, which shows that older adults can benefit from using streamlined platforms that are not only easy to use but may already be in use by the patients, so as to facilitate familiarization with the telemedicine platform adopted.^14^ Also, caregiver technical assistance as a facilitator to the use of telemedicine is a finding that is supported by other studies.^14,19,20^ Unsurprisingly, many older adults with dementia, cognitive impairment, or audiovisual problems have an even greater need for caregiver assistance than other older adults who do not have these concerns.^20^

By interviewing different groups of people and allowing them to share their perspectives on telemedicine use, we were able to obtain a rich source of data to inform our conclusions. Based on our findings, it does appear as if the barriers to telemedicine use far exceed the facilitators, and, as a result, our recommendations are geared toward reducing the identified barriers. Table 2 provides a summary of some of the barriers and facilitators and how they can be explained using the theory of planned behavior, while Table 3 lists some of the recommendations that were given by our participants for improving telemedicine video visits for older adults.

**Table 2.** Facilitators and Barriers

Facilitator (F) and barrier (B) themes according to the TPB Model	
TPB model component (example quotes)	Themes.
Attitude “And by the way, the positive for my father is that he's basically deaf and he does have two hearing aids, but he reads lips. And so, when you've got a mask on in the office, it's very hard for him to understand what's going on. But when he could see Dr. D's face right in the screen, that was easier for him”.—Informal Caregiver	• Provider sensitivity (ICG)—F • Removal of PV—F • Providing information about patient wait times (SC) —F • Happiness at avoiding the hospital building (PT)—F
“And I had some Parkinson's patients who, for whatever reason, I don't think they like me in their space as much, and so talking to a computer was less threatening and they seemed to open up a little bit. So, we have the full range of experience.”—Physician	
Subjective norm “And I know lots of people who did that. And I think that generally makes them familiar with communicating by an audio-visual method.” —Patient	• Supportive staff during visit (PT)—F • Using Zoom software for communication with others outside hospital (PT)—F • Spouse using telemedicine (PT) —F
“And I think that because we're technologically savvy, it really wasn't a big step for us. I mean, she and I, we each have and operate separate computers.”—Patient	
Perceived behavioral control “I mean, honestly, geriatric or not, that part is confusing for people to figure out how to download that third party app outside of MyChart”.—Scheduler “I think it's hard because these are end-stage Parkinson's and they're having trouble communicating, getting the words out. And so, they feel the pressure.”—Physician “The only time it is difficult, and we have a problem I think is when the patients can't figure it out and we're on the phone with them asking if they need help and we're not quite prepared on how to tell them what to do.”*—*MA “Oh, man, those help desk lines. I never think those work well. I guess it just depends on who's manning or womaning the desk.”—Informal caregiver “…well, a lot of the older people just don't understand it.”—Patient	• Rescheduled visit without prior notification (PT)—B • Help desk lines not always helpful (ICG)—B • App download difficulty (SC)—B • Patients' functional ability (PV)—B • Nonuser-friendly electronic record updates (SC)—B • Lack of staff telemedicine training (MA)—B • Device problems requiring switching (PV)—B • Difficulty reaching patients residing in a facility (SC)—B • Customer service support (MA)—F • Technology very easy to use (PT)—F • Straightforward log in process after first attempt (FCG)—F • Simplified process of patient consultation (PV)—F • Designing a stepwise protocol for patients (SC)—F
Intention “And honestly, I think a lot of geriatric patients, they're going to do it from their desktop or from a laptop anyway. I don't think a lot of them we had to talk about the telehealthConnect app.”—Scheduler	• Devices not requiring telehealthConnect app (SC)—F

PT, patient; ICG, informal caregiver; FCG, formal caregiver; PV, provider; SC, scheduler; TPB, theory of planned behavior.

**Table 3.** Areas of Improvement Identified by Participants

Participant category	Process improvement area	Actual quotes
Physician	Adding auxiliary equipment	“I've heard some of these systems can be more elaborate where they have stethoscopes and otoscopes and things that run right through the program, and so it can give more primary data. That might be useful, but obviously that comes at a cost.”
Patient	Using familiar video communication software	“And I know lots of people who did that [used other video technology software]. And I think that generally makes them familiar with communicating by an audio-visual method.”
Physician	Integrating telemedicine visits with medical records	“In terms of a medical records thing, yeah, you don't have the visit recorded or preserved, or the timing is not. They just have to take my word for it.”
Scheduler	Integrating the telehealthConnect app into MyChart	“Ideally, we would just have a video component built into MyChart, but right now it says, if you don't have this app downloaded, which is telehealth, you need to go download it from the app store. And then, MyChart will connect to the telehealth from there on out.”
Caregiver	Providing training and/or user-friendly technology	“But I just feel like there's a lot of us. older people are still learning about some of these technologies. Just more training, I guess or making some things more user friendly.”
	Reducing patients' technology anxiety by providing information	“And so, what I would say is that if people understand they only have to do it once and then it's resident on their computers, and this is the thing that they do to see their doctor as opposed to every time it's this drama, I think that would be really helpful.”
	Technology assistance	“Maybe you already have something set up or someone's sitting behind the screen all day long helping people log on. I don't know. But if you have it, that'd be great.”
MA	Providing training/technical support	“So, either have training for the MA to get them ready or have a readily available IT person that could help them get ready for it, because that was the biggest issue with any of this.”

In addition to identifying the barriers and facilitators, this study suggests ways to improve the use of telemedicine video visits in older patients. One recommendation that was given by a participant was to integrate the telehealth application into patients' electronic health record (MyChart). Due to the cumbersome process of downloading the app outside MyChart, linking the app to MyChart was assumed to be more efficient. This integration, if implemented with trainings, and as-needed technical support, were considered to be beneficial in improving the self-confidence of the end users. We therefore recommend that tailored training sessions be offered before end users start using telemedicine, with opportunities for testing and feedback provided. Also, ongoing technical support should be readily available, accessible, and easy to understand by end users.

As important as it is for health systems to be innovative, it is equally important for them to assess the efficiency of their innovations by listening to the voices of their end users and taking necessary action to improve their experiences. Our findings reinforce the need to involve older adults in the design of telemedicine systems, seeking their perspectives on an ongoing basis to know what their barriers and facilitators are, to ensure that their continued access to health care in the current pandemic and subsequent pandemics is not interrupted. Providing solutions to these barriers will promote adoption of telemedicine by this population, improve health outcomes, and provide a basis for the expansion of telemedicine by health care organizations.

### Limitations

Our small sample size of 33 participants might limit the generalizability of the results while our regional sample (Upper Midwest) might not be nationally representative. Also, the use of a cross-sectional design, while helpful, does not capture changes in experiences with telemedicine use over time. In addition, there is a chance that some social desirability bias might have been introduced by using the patients of the physicians at the Family Medicine clinic, as the patients might not feel comfortable revealing any provider-related negative experiences with telemedicine. Furthermore, with few participants in each category, the results could have been biased as our findings might not be representative of the larger potential population of participants.

## Conclusion

This study went a step further to suggest areas of improvement based on the everyday experiences of patients, caregivers, health care, and nonhealth care personnel at our facility. These end users of telemedicine technology shared information on how the telemedicine delivery process could be improved upon to increase efficiency in accessing health care for older adult patients—an important topic to consider in uncertain times like a pandemic and among vulnerable populations like older adults who already face a digital divide in health care access.^21^

Many digital platforms are often not tailored for use by the elderly, leading to utilization challenges. This stems from the fact that the perspectives of older adults about the usability of telemedicine technology are not often sought by system developers. As a result, there is often a poor fit between the telemedicine system, the task to be performed, and the older adult user who performs the task. With the increase in the population of older adults with chronic conditions and age-related disabilities, and the potential of another pandemic, it is imperative for health systems to proactively solicit the input of end users before innovative systems like telemedicine are rolled out, to ensure that their needs are anticipated, identified, and addressed on an ongoing basis.

This will not only ensure that the design is a good fit for them but will also identify and resolve potential barriers early, promoting the uptake of telemedicine among older adults and the scale up of telemedicine systems by health care organizations, making the sustainability of the telemedicine intervention possible.

Opportunities for future research focusing on system redesign and use may include comparing the barriers and facilitators that geriatric populations experience with those faced by younger populations.

It is hoped that the recommendations by the participants will lead to the implementation of interventions and the enactment of policies that not only improve access to telemedicine video systems but also enhance the telemedicine experiences of older adults.

## Abbreviations Used

MA: medical assistant
SLC: Senior Living Community
